# Correction: Impact of immune evasion, waning and boosting on dynamics of population mixing between a vaccinated majority and unvaccinated minority

**DOI:** 10.1371/journal.pone.0330965

**Published:** 2025-08-26

**Authors:** David N. Fisman, Afia Amoako, Alison Simmons, Ashleigh R. Tuite

After publication of this article [1], concerns were raised about the definition of ψ and undeclared competing interests. After editorial follow up and consultation with a member of *PLOS One*’s Editorial Board, the *PLOS One* Editors concluded that the article’s overall results and conclusions are upheld; this notice serves to address errors and omissions as follows:

- There is an error in the description of the ψ parameter in the third sentence of the second paragraph of the Analyses subsection of the Methods section. The correct definition of ψ appears in an earlier work [4], and in the caption to Fig 3 in this article [1]. The correct sentence is:

“As in our earlier work [4] we estimate a quantity that we denote ψ, defined as the ratio of the fraction of infections acquired by vaccinated people from unvaccinated people, divided by the fraction of contacts with unvaccinated people.”

As noted in the earlier publication, this represents “a normalized index of the degree to which risk in one group may be disproportionately driven by contact with another” [4]. The authors apologize for this error in the published article.

- The fourth paragraph of the Results section reports that “In univariable sensitivity analyses on disease natural history, vaccine efficacy and durability of response, and booster dose frequency, we found no change in qualitative model projections when parameter inputs were varied over plausible ranges.” The sensitivity analysis on disease natural history (basic reproductive number (R_0_)) was not included in the originally published Supporting Information; here, the authors provide these data in S1 File with this notice. As demonstrated in Supplementary Figure 5, R_0_ was varied over a wide range (from 2.5, approximating the R_0_ seen with initial SARS-CoV-2 emergence, to 12, an R_0_ seen with highly contagious diseases like measles, and likely approximating the R_0_ for the Omicron variants of SARS-CoV-2) [2,3]. For all values of R_0_ evaluated and across a range of assumptions related to population mixing, both the value of ψ and relative cumulative incidence among the unvaccinated were substantially greater than 1, indicating disproportionate contribution to risk of infection from unvaccinated individuals, as well as greater risk to unvaccinated individuals themselves.
- A Competing Interests statement was provided by the authors at the time of submission; however, an incomplete version of the article metadata which omitted these items was published in error. The publisher apologizes for this error. The correct Competing Interests statement is:

“Declaration of competing interests: DNF has served on advisory boards related to influenza and SARS-CoV-2 vaccines for Seqirus, Pfizer, AstraZeneca and Sanofi-Pasteur Vaccines, and has served as a legal expert on issues related to COVID-19 epidemiology for the Elementary Teachers Federation of Ontario and the Registered Nurses Association of Ontario. ART was employed by the Public Health Agency of Canada when the research was conducted. The work does not represent the views of the Public Health Agency of Canada.”

The authors provide the following additional discussion and clarification:

The original paper assumed that all individuals experience immune protection after infection, in contrast to a fraction of individuals receiving immune protection after vaccination (with that fraction dependent on estimated vaccine efficacy). Greater durability of protection from vaccination than natural infection was assumed, based on a meta-analysis and statistical modeling work by Townsend et al [5]. The editorial board member consulted on this paper noted that there is some evidence that immune protection may be more durable after infection than after vaccination [6–10]. To address this, additional analyses were performed, in which the initial model was re-parameterized, such that the rate of loss of immunity after vaccination was 1.5-times faster than after infection (rather than 25% slower, as in the base case). This results in no qualitative change to the model results or conclusions, for the simple reason that infections (and resultant infection-related immunity) occur in vaccinated individuals as well as unvaccinated individuals. This analysis is presented in Supplementary Figure 6 of the revised S1 File.

While the notion that immunity after infection is preferable to immunity after vaccination has become popular in some circles, it bears restating that the entire rationale for vaccination is to provide immunity without individuals having to experience infection, which is associated with morbidity and risk of mortality, and which is communicable to other people.

## Supporting information

S1 File Revised version of S1 Appendix in article [1]. (DOCX)
