# Supplementary material for: Impact of immune evasion, waning and boosting on dynamics of population mixing between a vaccinated majority and unvaccinated minority
Source: PLoS One. 2024 Apr 4;19(4):e0297093. doi: 10.1371/journal.pone.0297093 (PMC10994315; doi:10.1371/journal.pone.0297093)

**Supplementary Appendix**

An overview of the model is provided in the following model schematic. Full details are provided in the model equations below.

**Supplementary Appendix Figure 1. Stock and Flow Diagram of Transmission Model.**

Compartmental “SIR” (susceptible-infectious-recovered) compartmental model that incorporates history of infection in both vaccinated (denoted by the subscript “v”) and unvaccinated (subscript “u”) populations. The subscript 1 represents no prior history of infection; subscript 2 represents prior history of infection. Susceptible individuals transition to the infectious state at a rate defined by Σβ_ijk_f_ij_(I_jk_/N_jk_), where subscripts *i, j,* and *k* denote vaccination status of the group at risk, vaccination status of contacts and prior infection status of contacts respectively; β represents a transmission coefficient, f denotes mixing patterns derived from a mixing matrix, N represents population size, and δ diminished infectivity based on prior immune experience. Infectious individuals recover at a rate of γ and lose immunity at a rate of ζ.

**
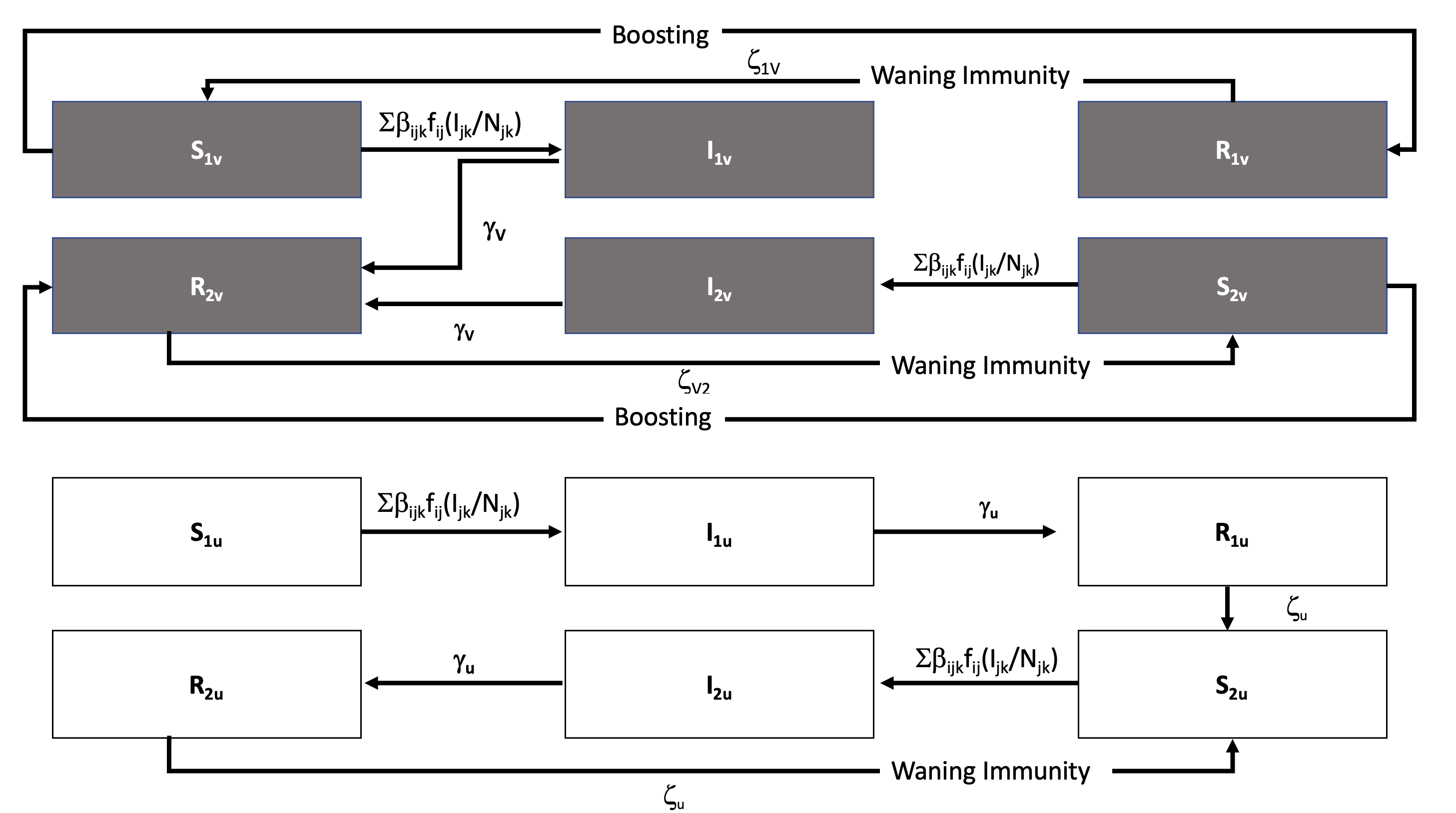
**

The epidemiology of disease transmission in vaccinated and unvaccinated subpopulations in the model is governed by the following ordinary differential equations, where subscripts 1 and 2 denote lack of prior infection or prior infection; subscripts U and V denote unvaccinated or vaccinated subpopulation. Subscripts *i, j,* and *k* denote vaccination status of the group at risk, vaccination status of contacts and prior infection status of contacts respectively. f denotes mixing patterns derived from a mixing matrix. Note that vaccination is initially represented by subdivision of vaccinated population into susceptible and immune categories based on vaccine efficacy. Boosting occurs via periodic pulses that move individuals from S🡪R classes within the vaccinated population, with that proportion again determined by vaccine efficacy.

*Unvaccinated Subpopulations*

dS_1U_/dt = - S_1U_Σβ_ijk_f_ij_(I_jk_/N_jk_) (1)

dI_1U_/dt = S_1U_Σβ_ijk_f_ij_(I_jk_/N_jk_) - γ_U_I_IU_ (2)

dR_1U_/dt = γ_U_I_IU_ - ζ_U_R_1U_ (3)

dS_2U_/dt = -S_2U_Σβ_ijk_f_ij_(I_jk_/N_jk_) + ζ_U_ R_2U_ (4)

dI_2U_/dt = S_2U_Σβ_ijk_f_ij_(I_jk_/N_jk_) - γ_U_I_2U_ (5)

dR_2U_/dt = γ_U_I_2U_ - ζ_U_R_2U_ (6)

*Vaccinated Subpopulations*

dS_1V_/dt = - S_1V_Σβ_ijk_f_ij_(I_jk_/N_jk_) + ζ_1V_R_1V_ (1)

dI_1V_/dt = S_1V_Σβ_ijk_f_ij_(I_jk_/N_jk_) - γ_V_I_IV_ (2)

dR_1V_/dt = −ζ_1V_R_1V_ (3)

dS_2V_/dt = -S_2V_Σβ_ijk_f_ij_(I_jk_/N_jk_) + ζ_2V_R_2V_ (4)

dI_2V_/dt = S_2V_Σβ_ijk_f_ij_(I_jk_/N_jk_) - γ_V_I_2V_ (5)

dR_2V_/dt = γ_V_(I_1V_+I_2V_) - ζ_V2_R_2V_ (6)

**Supplementary Appendix Figure 2. Two-Way Sensitivity Analysis on Durability of Immune Protection**

Plots of cumulative value of ψ (A) and cumulative relative risk of infection among the unvaccinated (B) with variation in the hazard ratio for loss of immune protection among vaccinated people, relative to unvaccinated people with post-infection immunity (X-axis). Colored curves represent different hazard ratios for loss of immune protection in vaccinated individuals with prior infection (so-called “hybrid immunity”); hazard ratios for hybrid immunity are presented in legends. Note that hazard ratios are multiplicative, such that hazard ratios for loss of hybrid immunity are relative to loss in individuals with a history of vaccination and no prior infection.
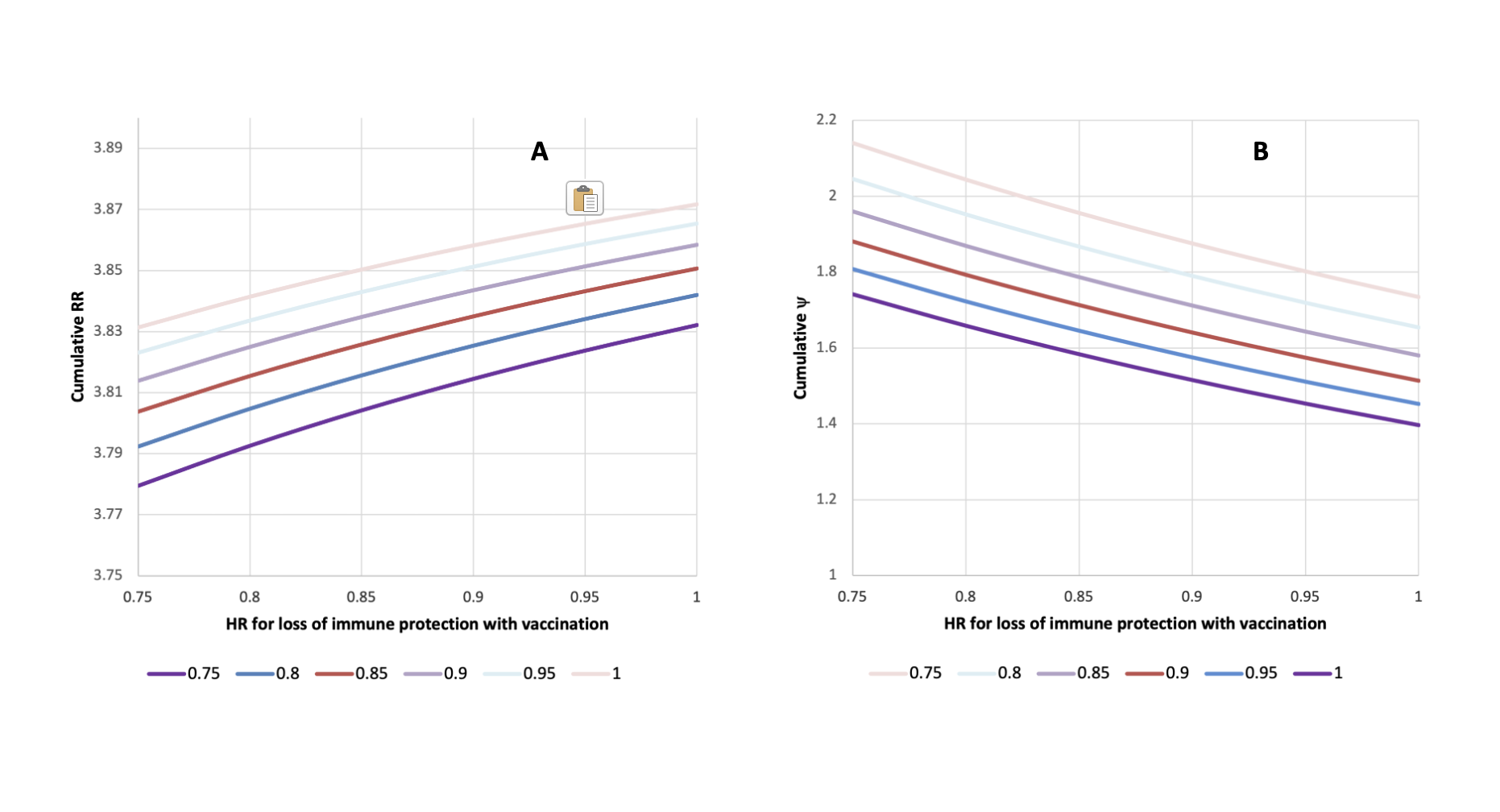


**Supplementary Appendix Figure 3. Two-Way Sensitivity Analysis on Initial Vaccine Efficacy and Booster Frequency**

Plots of cumulative value of ψ (A) and cumulative relative risk of infection among unvaccinated people (B) with variation in interval between booster vaccine doses (X-axis). Colored curves represent initial vaccine efficacy estimates (as presented in legends).


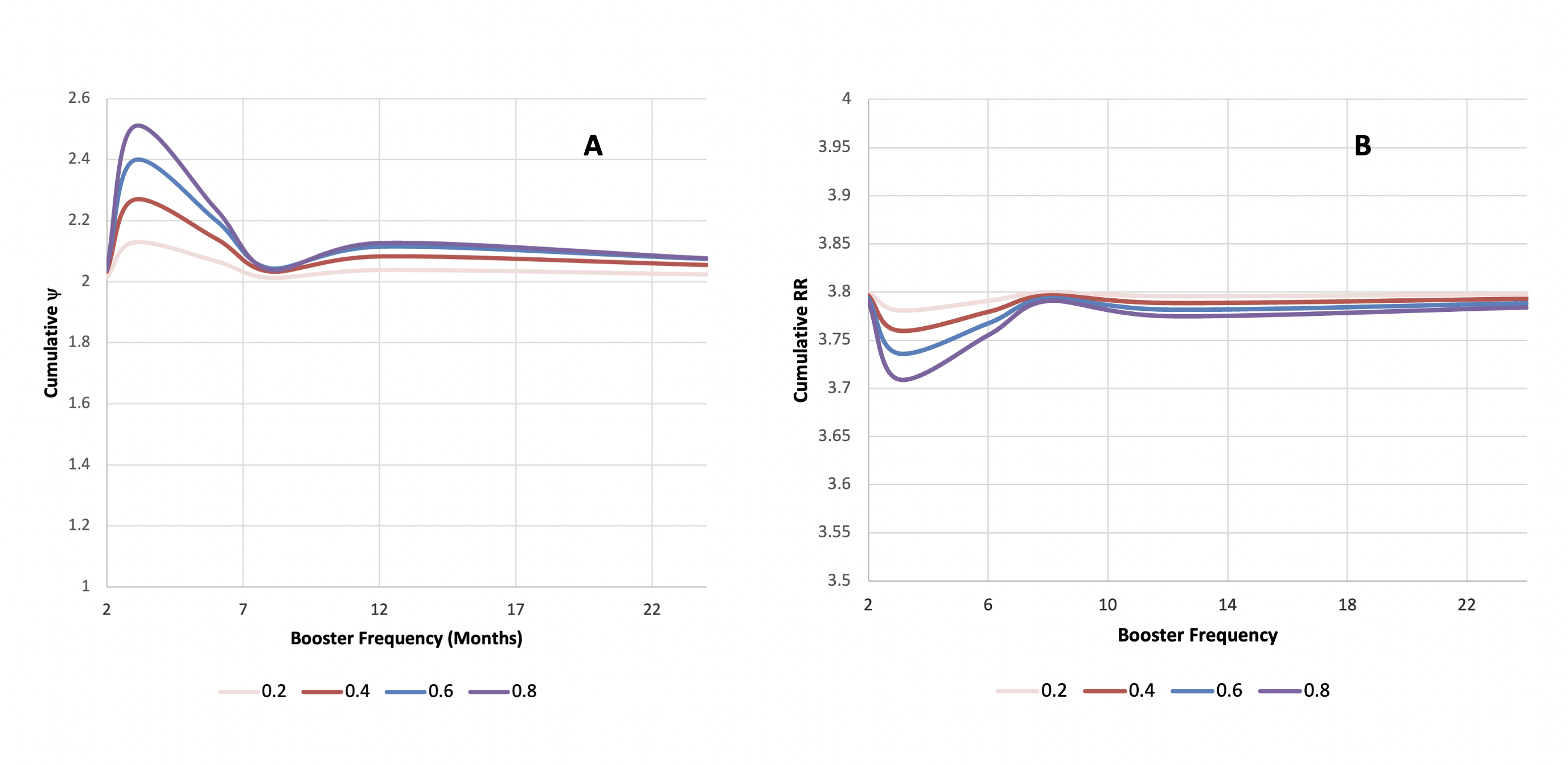


**Supplementary Appendix Figure 4. Two-Way Sensitivity Analysis on Impact of Prior Infection and Vaccination Status on Infectivity**

Plots of cumulative value of ψ (A) and cumulative relative risk of infection among the unvaccinated (B) with variation in reduction in infectivity by prior vaccination or infection (X-axis). Colored curves represent risk reduction associated with prior infection in vaccinated individuals (so-called “hybrid immunity”) (as presented in legends).


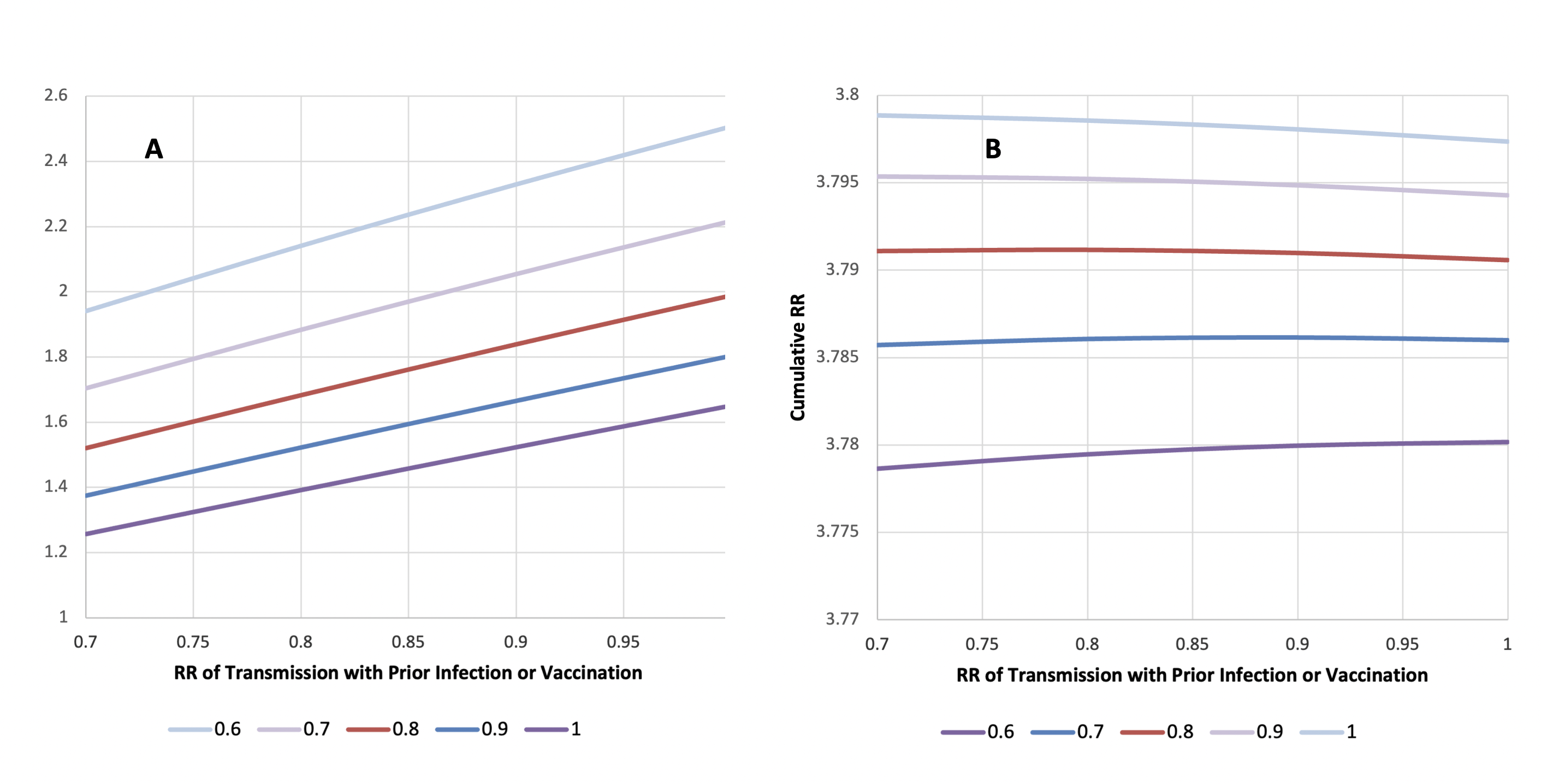

Supplement: S1 Appendix — (DOCX) [file pone.0297093.s001.docx]
